# β-*N*-Oxalyl-l-α,β-diaminopropionic Acid (β-ODAP) Content in *Lathyrus sativus*: The Integration of Nitrogen and Sulfur Metabolism through β-Cyanoalanine Synthase

**DOI:** 10.3390/ijms18030526

**Published:** 2017-02-28

**Authors:** Quanle Xu, Fengjuan Liu, Peng Chen, Joseph M. Jez, Hari B. Krishnan

**Affiliations:** 1 College of Life Sciences, Northwest A&F University, Yangling 712100, Shaanxi, China; xuql03@163.com (Q.X.); liufengjuan2015@163.com (F.L.); pengchen@nwsuaf.edu.cn (P.C.); 2 Department of Biology, Washington University in St. Louis, St. Louis, MO 63130, USA; jjez@wustl.edu; 3 Plant Genetics Research Unit, USDA-Agricultural Research Service, 108 Curtis Hall, University of Missouri, Columbia, MO 65211, USA

**Keywords:** β-cyanoalanine synthase, β-ODAP, *Lathyrus sativus*, nitrogen, sulfur

## Abstract

Grass pea (*Lathyrus sativus* L.) is an important legume crop grown mainly in South Asia and Sub-Saharan Africa. This underutilized legume can withstand harsh environmental conditions including drought and flooding. During drought-induced famines, this protein-rich legume serves as a food source for poor farmers when other crops fail under harsh environmental conditions; however, its use is limited because of the presence of an endogenous neurotoxic nonprotein amino acid β-*N*-oxalyl-l-α,β-diaminopropionic acid (β-ODAP). Long-term consumption of *Lathyrus* and β-ODAP is linked to lathyrism, which is a degenerative motor neuron syndrome. Pharmacological studies indicate that nutritional deficiencies in methionine and cysteine may aggravate the neurotoxicity of β-ODAP. The biosynthetic pathway leading to the production of β-ODAP is poorly understood, but is linked to sulfur metabolism. To date, only a limited number of studies have been conducted in grass pea on the sulfur assimilatory enzymes and how these enzymes regulate the biosynthesis of β-ODAP. Here, we review the current knowledge on the role of sulfur metabolism in grass pea and its contribution to β-ODAP biosynthesis. Unraveling the fundamental steps and regulation of β-ODAP biosynthesis in grass pea will be vital for the development of improved varieties of this underutilized legume.

## 1. Introduction

**Figure 1:**
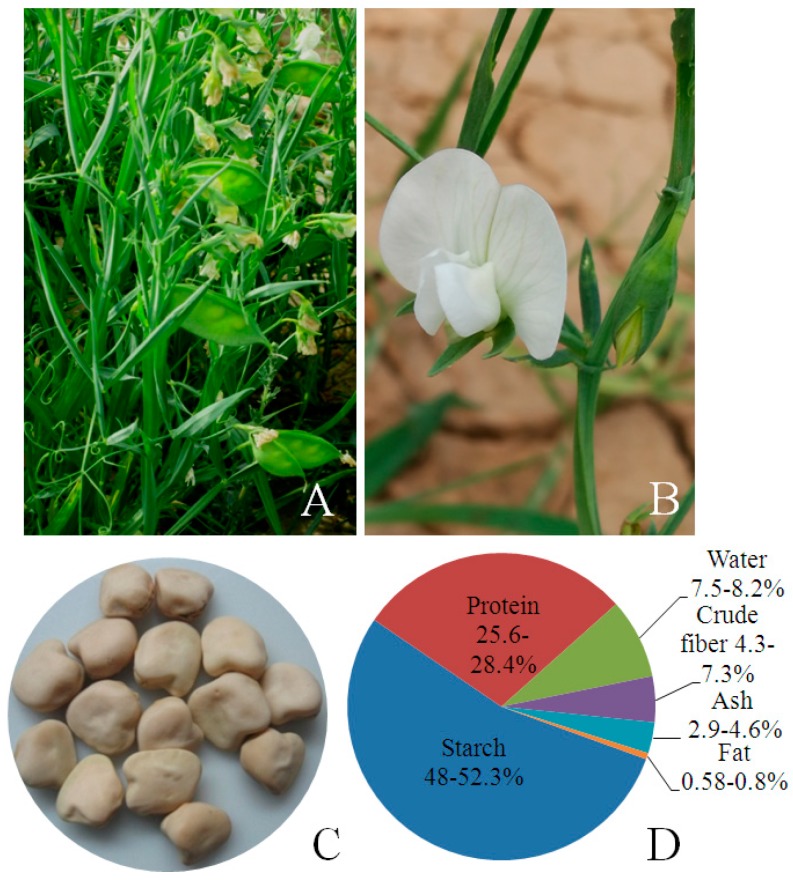
*Lathyrus sativus* (grass pea), an annual legume cultivated in arid and semiarid areas, has attractive flowers (**A**,**B**) and yields nutritious seeds (**C**). The seeds are a rich source of protein and starch (**D**).

The genus *Lathyrus* includes about 187 species and occurs both in the Old World and the New World [1]. The members of this genus are resistant to biotic and abiotic stress such as insects and pests, drought, water logging, salinity, and low soil fertility [1,2,3,4]. They produce attractive blue, pink, red, and white colored flowers as well as flowers with assorted combinations of these colors (Figure 1A,B). Some species, such as *L. odoratus*, are grown for their ornamental value. Among them, grass pea (*L. sativus* L.) is widely cultivated as an edible and forage crop in North Africa, Near East, western Asia, and the Indian subcontinent [1,3,4]. The deep penetrating root systems of *Lathyrus* species enables them to thrive in drought conditions. Additionally, they form a symbiotic association with soil rhizobia, which allows for fixation of atmospheric nitrogen. Biological nitrogen fixation, in addition to minimizing the use of fertilizers, also positively impacts the soil nitrogen balance. Grass pea is notable for its high protein content and its seeds are an inexpensive source of highly nutritious and well-balanced human dietary protein [1,4] (Figure 1C). The seed contains about 28% protein, 48% starch, and less than 1% fat (Figure 1D). Like most other legumes, grass pea seed contains low amounts of sulfur-containing amino acids (cysteine and methionine), but has relatively high amounts of lysine and threonine [1,4,5].

Although grass pea has desirable traits, the use of grass pea is limited by the presence of a neurotoxic non-proteinogenic amino acid β-*N*-oxalyl-l-α,β-diaminopropionic acid (β-ODAP). Consumption of grass pea as a main or sole diet for several months causes lathyrism, a neurodegenerative syndrome that results in the paralysis of lower limbs [6,7,8]. β-ODAP is found in all parts of the plant with the highest content reported in the leaf at vegetative stage and in the embryo at the reproductive stage [9,10]. The accumulation of β-ODAP is influenced by the environment and growing conditions, and its levels in different grass pea lines can range from 0.22 to 7.20 g/kg [3,11,12]. Multiple investigations of β-ODAP-induced lathyrism indicate that β-ODAP has neurotoxic potential [6,7,13,14], however, its causative role in lathyrism still remains to be proven [15].

For the development of safer grass pea varieties, concerted research efforts to understand the mechanism of lathyrism, to elucidate the β-ODAP biosynthetic pathway, and to understand how different environmental factors and growing conditions affect β-ODAP content are needed. Initial work suggests that nitrogen supply plays a major role because it dramatically affects β-ODAP content [10,16]. Diet also influences the development of lathyrism, as consumption of *L. sativus* mixed with vegetables rich in sulfur amino acids like onion and garlic has been shown to have a protective effect against neurolathyrism [17,18]. This result implies that dietary deficiency of sulfur-containing amino acids may contribute to the progression of lathyrism [19,20,21]. Moreover, the uptake of β-ODAP into nerve cells is inhibited by the presence of cysteine [22]. For these reasons, the development of safer grass pea genotypes should not only focus on lowering the β-ODAP content, but also increasing the content of sulfur-containing amino acids at the same time. Therefore, investigating the possible relationship between β-ODAP accumulation and sulfur metabolism is essential for the development of safer *L. sativus* lines.

## 2. Role of β-ODAP in Plants

The occurrence of β-ODAP has been reported in 21 *Lathyrus* species, 17 *Acacia* species, 13 *Crotalaria* species [23], and several other non-legume plant species [24,25,26,27]. Generally, β-ODAP in plants is hypothesized to function as a carrier molecule for zinc ions [28], a scavenger for hydroxyl ions [29], and as a protector of photosynthesis at high light intensity [30]. Some studies suggest that β-ODAP also plays a role in drought tolerance and in resistance to oxidative stress [31,32]. Currently, our knowledge on the biological role of this important metabolite is far from complete. Until we have a better understanding of the role of β-ODAP in plants, the goal of its complete elimination in grass pea should be approached with caution.

## 3. Genetic Studies of *L. sativus* Breeding

Developing improved varieties of *L. sativus* with lowered β-ODAP content has been a goal of plant breeders. Evaluation of grass pea germplasm reveals a wide range (0.02%–2.59% of seed weight) of β-ODAP content [3]; however, no β-ODAP-free lines have been identified in either grass pea germplasm or wild *Lathyrus* species [3,33,34]. Evaluation of 1082 accessions of grass pea identified four lines with low ODAP content ranging from 0.007%–0.02% of seed weight [3].

Interestingly, grass pea lines with low ODAP content were compromised in many agronomic traits, which suggests that ODAP may play a role in plant growth and development [3,35]. Through conventional hybridization, mutant breeding, and somaclonal variation several low β-ODAP lines have been released in India and Ethiopia [3]. Multi-location yield trials show that these improved lines also maintained high yield. For example, one of the *L. sativus* lines developed by ICARDA (International Center for Agricultural Research in the Dry Areas) yielded 1.67 ton·ha^−1^ and had low ODAP content of 0.08% of seed weight [3]. β-ODAP content is affected by genotype and external environment factors, such as water stress, salinity, drought, and exposure to heavy metals [3,36,37,38,39]. Even grass pea cultivars with low β-ODAP content showed considerable variability when these cultivars were grown under different environmental conditions [3].

Currently, the number of genes controlling β-ODAP content and the enzymes responsible for the biosynthesis of β-ODAP is not precisely known. To date, the genetics of β-ODAP content has received only limited attention. Tripathy et al. [38] studied the genetics of β-ODAP content by crossing different *L. sativus* varieties with different β-ODAP levels. They found significant difference among parents and crosses for mean β-ODAP content and concluded that there were more than two genes or loci involved in the biosynthesis of β-ODAP in seeds [38]; however, the key genes involved in the metabolism of β-ODAP remain to be identified [39].

## 4. β-ODAP Biosynthesis in Grass Pea

**Figure 2:**
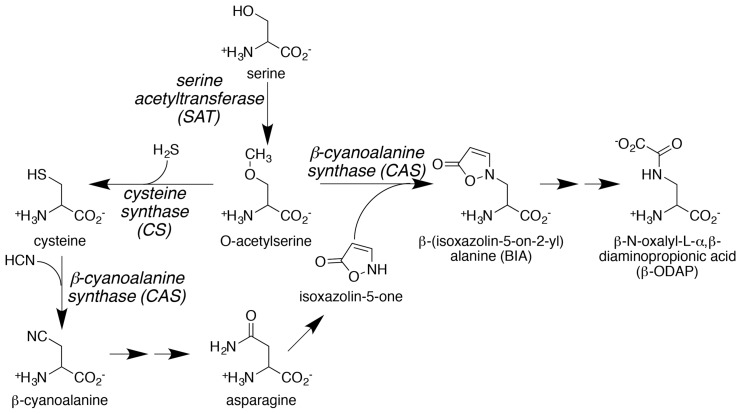
β-*N*-oxalyl-l-α,β-diaminopropionic acid (β-ODAP) biosynthetic pathway in grass pea. Reactants and products of the pathway are shown in normal font and enzymes associated with the pathway are italicized.

The enzymatic pathway of β-ODAP biosynthesis is not fully understood [40,41]. It is believed that its synthesis begins with formation of β-(isoxazolin-5-on-2-yl)alanine from reaction of *O*-acetylserine and isoxazolin-5-one (Figure 2). Early studies suggest that β-cyanoalanine synthase (CAS) uses isoxazolin-5-one as an alternative nucleophile in the reaction [40,41]. Next, β-(isoxazolin-5-on-2-yl)alanine (BIA) is proposed to be converted to the short-lived intermediate 2,3,-l-diaminopropanoic acid, which is subsequently oxalylized by oxalyl-coenzyme A to form β-ODAP [42,43,44,45]. Although high concentrations of β-(isoxazolin-5-on-2-yl)alanine have been reported in the seedlings of grass pea, garden pea, and lentil, the occurrence of 2,3-l-diaminopropanoic acid has not been verified [21].

Production of isoxazolin-5-one is essential for β-ODAP synthesis and appears linked to the normal metabolic activities of cysteine synthase (CS; also known as either *O*-acetylserine sulfhydrylase or *O*-acetylserine(thiol)lyase) and CAS [46,47]. In cysteine biosynthesis, CS catalyzes cysteine formation from O-acetylserine and hydrogen sulfide. Cysteine is then used as a substrate for CAS in the detoxification of cyanide in plants [48]. Subsequent conversion of β-cyanoalanine to asparagine leads to the generation of isoxazolin-5-one. Thus, β-ODAP content in *L. sativus* is controlled via the integration of nitrogen and sulfur metabolism through the biochemical activities of CAS.

Several lines of evidence suggest that the neurotoxicity of β-ODAP is related to the content of sulfur amino acids methionine and cysteine. Getahun et al. [17,18] demonstrated that consumption of grass pea in combination with vegetables rich in sulfur-containing amino acids lowered the neurotoxicity of β-ODAP. Depletion of methionine and cysteine in the growth medium also aggravates the neurotoxicity of β-ODAP to isolated neurons [19]. Human lathyrism may also be related to the oxidative stress caused by the absence of sulfur-containing amino acids [49,50]. The low levels of methionine and cysteine in grass pea may be at least equally important in the etiology of neurolathyrism as the presence of β-ODAP [50]. Therefore, it was proposed that increasing the sulfur-containing amino acids in grass pea might help prevent neurolathyrism, even without substantially lowering levels of or eliminating β-ODAP [39,50].

## 5. Biosynthesis of β-ODAP in *L. sativus* Is Related to Sulfur Metabolism

The biosynthesis of β-ODAP is connected to the sulfur amino acid biosynthetic pathway (Figure 2) [46,47]. β-ODAP is derived from heterocyclic β-(isoxazolin-5-on-2-yl)alanine, formed by a reaction catalyzed by CAS [47]. The direct precursor α,β-diaminopropionic acid is enzymatically formed from β-(isoxazolin-5-on-2-yl)alanine [44,45]. Interestingly, both the formation of cysteine and β-(isoxazolin-5-on-2-yl)alanine compete for the same substrate (i.e., *O*-acetylserine) (Figure 2). In these reactions, isoforms of the β-substituted alanine synthase (BSAS) family of enzymes play critical roles. The BSAS family of proteins, which includes CS and CAS, catalyze pyridoxal phosphate-dependent synthesis reactions [51,52]. Biochemical studies of proteins isolated from *L. sativus* identified BSAS-like enzymes that catalyzed the formation of β-(isoxazolin-5-on-2-yl)alanine [46,47,53]. For example, Ikegami et al. [46,47] isolated two different forms of CS from *L. sativus*. Both enzymes functioned as dimeric proteins with a subunit molecular weight of 35 (CS-A) and 39 kDa (CS-B), respectively. Interesting, both isoenzymes displayed similar catalytic activity in converting *O*-acetylserine OAS to β-(isoxazolin-5-on-2-yl)alanine in spite of differing *K*_m_ values for OAS [46,47,54]. The efficiencies of the two isoenzymes to catalyze the formation of β-(isoxazolin-5-on-2-yl)alanine were less than 0.1% of the specific activity observed for the formation of cysteine from OAS and H_2_S [47]. Currently, it is unclear if formation of β-(isoxazolin-5-on-2-yl)alanine is a side-reaction of either CS or CAS or is catalyzed by a BSAS isoform that remains to be isolated. Recently, Jiao et al. [55] reported the isolation of CS isoforms from grass pea and their relationship to seed β-ODAP content.

## 6. Cysteine Synthase (CS) and β-Cyanoalanine Synthase (CAS) in *L. sativus*

CS is a key regulatory enzyme involved in cysteine biosynthesis in plants. Cysteine biosynthesis involves the acetylation of serine by acetyl-CoA generating OAS, a step catalyzed by serine acetyltransferase (SAT). CSase catalyzes the next step, which involves the β-replacement of the acetyl group of *O*-acetylserine with sulfide, resulting in the production of cysteine and sulfide (Figure 2). This reaction parallels CAS, which synthesizes β-cyanoalanine from cyanide and cysteine. In all plant species that have been examined so far, a small multigene family encodes CS, CAS, and related enzymes. For example, in *Arabidopsis*, there are nine *BSAS* genes that encode multiple CS and CAS isoforms [56]. In grass pea, five BSAS isozymes (I–V) were detected by native-polyacrylamide gel electrophoresis [55]. Based on enzyme activity it was suggested that isozyme I was CAS, while the others (II–V) were classified as CS. The temporal and tissue-specific accumulation of these isozymes was also investigated. The electrophoretic band corresponding to isozyme I (CAS) was detected in the cotyledons from two-day-old seedlings with protein content increasing during seed germination in different tissues. The maximum accumulation was detected in the taproots of four-day-old seedlings. The content of CAS decreased in most tissues of eight-day-old seedlings. This temporal variation of CAS protein matches the accumulation pattern of β-ODAP during grass pea seed germination. The highest level of β-ODAP accumulation occurred in young six-day old seedlings followed by a drastic decrease in older seedlings [10].

Recent work from the T-DNA insertion mutants of the mitochondrial β-cyanoalanine synthase (*CAS-C1*) showed that CAS is essential for root hair development in *Arabidopsis* [57,58]. If CAS plays a similar role in grass pea, then changes in CAS might affect the formation of root nodules [16]. Interestingly, it was reported that the five-day-old seedlings of *L. sativus* inoculated with *Rhizobium* contained lower levels of β-ODAP than the controls, and that nitrogen deficiency caused the highest accumulation of β-ODAP in the seedlings [10,32]. Studies have shown that CS plays an important role in linking sulfur and nitrogen assimilatory pathways and regulating the flux between these two pathways [59,60]. Takahashi and Saito (1996) [60] reported a five-fold increase in mRNA level of CysC (a mitochondrial isoform of CS in spinach) under either nitrogen deficient or both nitrogen and sulfur deficient conditions. The authors suggested that this might be due to a requirement for detoxification of excess amounts of sulfide, presumably released by the breakdown of sulfur-containing storage compounds [60].

Published data suggest that β-ODAP is synthesized in mitochondria and chloroplasts [46,47]. Interestingly, the same organelles also play an important role in cysteine biosynthesis [61,62]. It was reported that increased expression of genes encoding CS could result from elevated thiol levels. In plants, a key biochemical control feature of sulfur metabolism involves the association of CS with serine acetyltransferase (SAT), resulting in the formation of cysteine regulatory complex (CRC) [63]. The CRC acts as a sensor to coordinate plant sulfur metabolism and control cysteine production in plants [56,63,64,65,66]. In spite of the importance of sulfur metabolism in cysteine and β-ODAP production in grass pea, we know very little about the proteins involved in their biosynthesis. Manipulation of critical enzymes related to the sulfur assimilation pathway is thought to be the most promising approach for increasing the content of sulfur amino acids [67]. Given the importance of sulfur metabolism in the biosynthesis of β-ODAP, a complete understanding of the role of key sulfur assimilatory enzymes that regulate the biosynthesis of β-ODAP is essential.

## 7. β-Cyanoalanine Synthase (CAS) and Cyanide Detoxification in *L. sativus*

Cyanide (CN^−^ is a naturally occurring molecule in plants and is produced during ethylene biosynthesis [48]. It also acts as a signaling molecule in regulating metabolic processes like seed germination and dormancy release [68], and plays a role in resistance to biotic and abiotic stresses [69,70]. Plants can use cyanide for nitrogen transport, or nitrogen storage, or as reservoir for defense against herbivores. Cyanide inhibits cytochrome c oxidase in the mitochondrial electron transport system and metalloenzymes [48].

Cyanide detoxification is mediated by mitochondrial localized CAS, which catalyzes the addition of CN^−^ to cysteine and yields β-cyanoalanine [71,72]. Several reports suggest that CAS serves as a metabolic bridge that links cyanide and cyanogenic compounds to primary nitrogen metabolism in plants [72]. It should be pointed out that nitrogen nutrition has a pronounced influence on β-ODAP content in *L. sativus* [10,16]. In spite of the important role of CAS in diverse biological functions, including the integration of nitrogen and sulfur metabolism, very little is known about this key enzyme in grass pea.

## 8. Molecular Cloning of β-Cyanoalanine Synthase (CAS) from *L. sativus*

**Figure 3:**
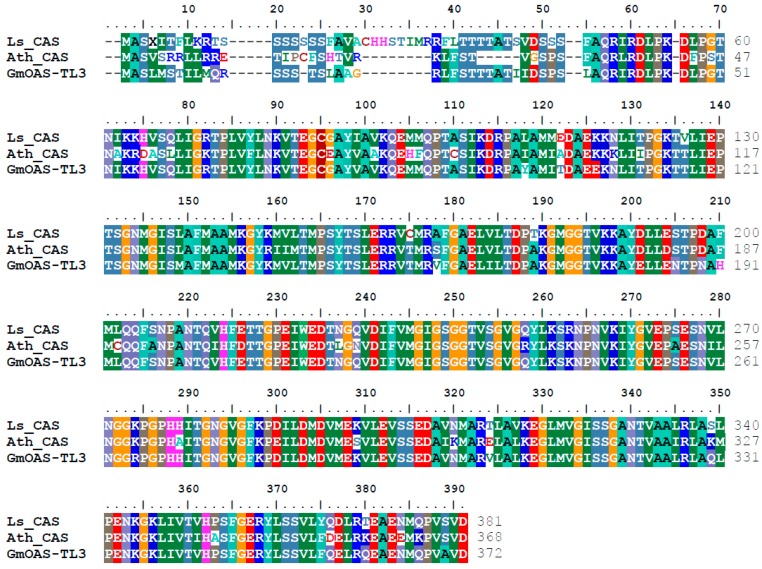
Amino acid sequence alignment of CAS. Protein sequences of grass pea (LsCAS), *Arabidopsis* (AthCAS) and soybean (GmOAS-TL3) were aligned using DNAMAN program version 8.0 (Lynnon LLC., CA 94583, USA).

**Figure 4:**
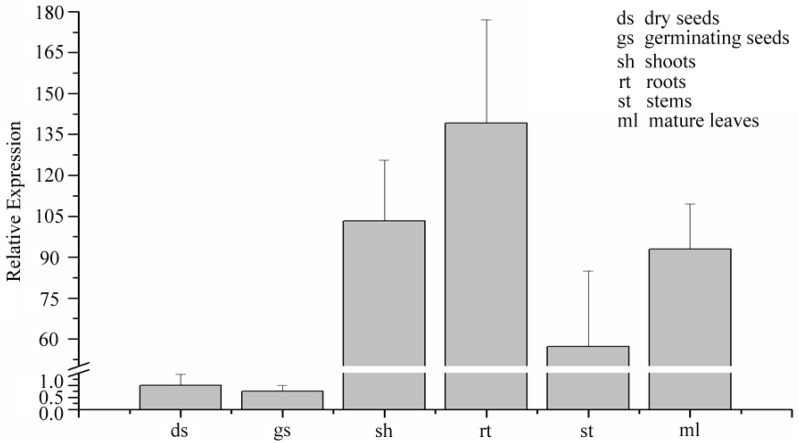
Quantitative reverse transcription-polymerase chain reaction analysis of *Ls-CAS* gene in different tissues of *L. sativus*.

For the development of nutritious and β-ODAP-free grass pea cultivars by genetic engineering, it is necessary to understand the contribution of the two key enzymes, CS and CAS, which catalyze the formation of cysteine and β-(isoxazolin-5-on-2-yl)alanine, respectively, for the synthesis of β-ODAP. Interestingly, these two enzymes also compete for the same substrate *O*-acetylserine (Figure 2). Recently, we have cloned the *CAS* gene from *L. sativus* (GenBank: KJ563188). Elucidation of the nucleotide and the derived amino acid sequence of the grass pea *CAS* gene showed extensive sequence homology with CAS from *Glycine max* and *Arabidopsis thaliana* (Figure 3). Grass pea CAS shared 88% and 79% amino acid sequence identity with soybean OAS-TL3 and *Arabidopsis* CAS, respectively (Figure 3). Similar to other CAS from other plant species, grass pea CAS is also a pyridoxal phosphate-dependent enzyme and is a member of the BSAS enzyme family. The deduced monomer molecular weight of grass pea CAS is 41 kDa, and the sequence encodes a mitochondrial localization sequence at the N-terminus of the protein. Analysis of the spatial-temporal pattern of the *CAS* gene shows expression in all the tissues that were examined, with the highest levels observed in the shoots and roots of young seedlings (Figure 4). Our observation is consistent with earlier biochemical studies reporting high levels of CAS enzyme activity in the roots and shoots of two to four day old seedlings [16]. Based on these observations, CAS may have a major role in regulating the β-ODAP content and its manipulation by genetic engineering presents a viable option for lowering β-ODAP content in grass pea.

**Figure 5:**
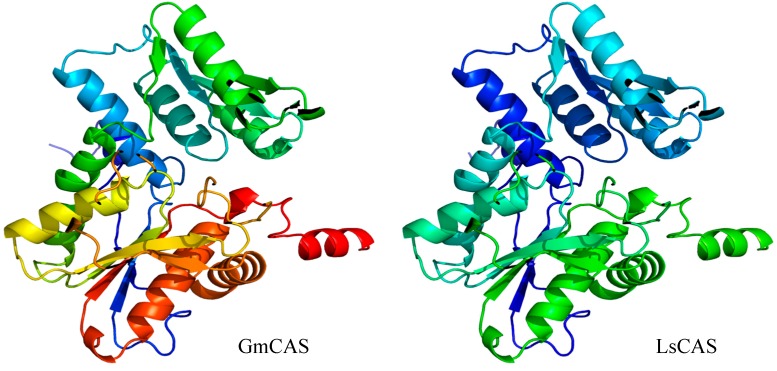
Homology-modeled structure of grass pea CAS. The 3-D structure of soybean [74] was used as a template to build a homology model of grass pea CAS using the Swiss-Model website (https://swissmodel.expasy.org) and viewed via Pymol.

Recently, the three-dimensional structure of soybean CAS (GmCAS) was elucidated by X-ray crystallography [73]. Because of the high sequence homology (93% identity), the crystal structure of GmCAS provides a template to homology model the grass pea CAS (LsCAS). LsCAS is predicted to share a conserved three-dimensional fold with GmCAS and the CS from *Arabidopsis* [74] (Figure 5). All these enzymes functions as dimeric pyridoxal phosphate-dependent members of the BSAS enzyme family. The PLP-attachment site in GmCAS has been identified as Lys95 [68,69]. Yi et al. (2012) [73] created mutants at this site (K95A) and compared its crystal structure to wild-type GmCAS. They found this mutation results in an alteration in the active site structure. We have generated individual mutants at this site (LsCAS K95A, K95E, and K95R) and found that these alterations resulted in almost a complete loss of CAS activity (unpublished data). These observations indicate that LsCAS K95A, K95E, and K95R mutations likely prevent binding of pyridoxal phosphate to impair catalytic activity. Sequence alignment reveals that Met-27 is highly conserved in CAS from different plant species, while this residue is replaced with Thr in AtCS (Figure 3). Interestingly, a triple mutant (T81M, S181M, and T185S) of GmCS resulted in the switching of the cysteine synthase activity to that of CAS [73].

Understanding the function of key sulfur assimilation and metabolism enzymes in grass pea may also contribute to efforts aimed at improving the overall sulfur amino acid content of this crop. An important feature of cysteine biosynthesis is the formation of a protein complex between serine acetyltransferase (SAT) and CS, which acts as a molecular sensor of intercellular sulfur conditions, thereby regulating the cysteine biosynthesis [56,63,64,65,66,74,75,76]. At least three *SAT* and five *CS* genes are found in *L. sativus* [56]. We have examined the protein-protein interaction between LsSAT1;1, LsSAT2;1. and LsSAT3;1 and LsCAS Our preliminary data reveal the interaction of LsSAT2;1 and LsSAT3;1 with LsCAS, while LsSAT1;1 did not interact. Recently, we have complemented an auxotrophic *E. coli* NK3 with the LsCAS gene, confirming that this gene functions in the biosynthesis of cysteine (unpublished data). To elucidate the precise function of grass pea CAS, efforts to silence the *CAS* gene expression, which may block β-ODAP synthesis, are in progress [77]. This approach may aid in the development of improved *L. sativus* lines with low β-ODAP and high sulfur-containing amino acids, thereby enabling wider cultivation of grass pea that is safer for human and animal consumption.

## 9. Oxidative Stress and β-ODAP Content in *L. sativus*

Oxidative stress is a serious imbalance between production of reactive oxygen species (ROS) and antioxidant defenses, which determines the extent of oxidative damage in the plant [78]. Reduced glutathione is one of the highly efficient antioxidant defense metabolites. Oxidative stress plays a crucial factor in neurolathyrism and other neurodegenerative diseases [17,18,20,21]. The deficiency of sulfur-containing amino acids cysteine and methionine, which can be considered as antioxidants, would contribute to oxidative stress. Experiments using cultured cells indicate that depletion of methionine and cysteine in medium aggravated the neurotoxicity of β-ODAP [19]. This observation suggests that the intake of cysteine and perhaps other antioxidants could help counter the effect of oxidative stress and aid in the prevention of neurolathyrism [19].

Mitochondrial dysfunction, which is a consequence of thiol oxidation caused by ROS (mainly superoxide anion (O_2_^.−^) and hydrogen peroxide (H_2_O_2_), is thought to be the primary cause of neurolathyrism [79]. In fact, ROS play important roles in the responses of plants to biotic and abiotic environmental stimuli, such as nutrient deficiency, drought, salinity, cadmium, and *Rhizobium* infection [16]. Leaves containing high levels of β-ODAP had low levels of O_2_^.−^ and H_2_O_2_, while leaves with high contents of O_2_^.−^ and H_2_O_2_ accumulated little β-ODAP [32]. This result suggested that ROS, especially O_2_^.−^, may either inhibit the synthesis of β-ODAP or enhance its degradation [16,32]. Interestingly, inoculation of *Rhizobium* to roots of young seedlings, which improves the nitrogen status of the plant, reduced β-ODAP contents in shoots [10] and enhanced both O_2_^.−^ and H_2_O_2_ levels [32]. This observation suggests a link between nitrogen availability and β-ODAP accumulation. In grass pea, the higher accumulation of β-ODAP occurred mainly in the very young seedlings and the ripening seed [10,11,12]. It was hypothesized that young tissues generally contain low levels of ROS, so the *O*-acetylserine may be used for the synthesis of BIA leading to the production of β-ODAP. While in mature leaves, which generally contain high levels of ROS, OAS may mainly be used to form cysteine [32] and subsequently glutathione, which are the key metabolites involved in antioxidant processes when ROS levels are very high [80]. However, the concentration of different metabolites (cysteine, glutathione ROS, and BIA) need to be quantified in these tissues to substantiate this hypothesis. Further investigation is required to establish the relationship between ROS and β-ODAP accumulation.

## 10. Role of Hydrogen Sulfide in Cysteine and β-ODAP Biosynthesis in *L. sativus*

Cysteine is the first committed molecule in sulfur metabolism that contains both sulfur and nitrogen, and thus, its metabolic regulation is of utmost importance for the synthesis of a number of essential metabolites, including β-ODAP [81]. As pointed out earlier, CAS catalyzes the conversion of cysteine and cyanide to β-cyanoalanine and H_2_S. In addition to β-CAS, two other enzymes, l-/d-cysteine desulfhydrase (l-/d-DES) and sulfite reductase (SiR) are also responsible for H_2_S synthesis. A balance between cysteine biosynthesis and its degradation to hydrogen sulfide (H_2_S) may be of significance not only in maintaining cysteine-homeostasis, but also in regulating sulfide-status and its possible effects on the antioxidant defense of plants experiencing stress [82]. Recent studies show that endogenously produced H_2_S may have a signaling function [83,84]. H_2_S regulates a wide range of physiological processes, including seed germination, abiotic stress tolerance, and senescence [85,86]. Furthermore, H_2_S competes with ROS and NO in thiol modifications of proteins in plants [87,88]. Since CS and CAS can regulate H_2_S homeostasis, its role in the biosynthesis of β-ODAP in grass pea requires in-depth investigation. 

## 11. Future Studies 

Considering the key role of sulfur in β-ODAP accumulation, the following aspects of research may become particularly important in the future:

♦ The biosynthetic pathway of β-ODAP has not yet been fully elucidated at present. A better insight into the biochemical and molecular mechanisms for β-ODAP biosynthesis is urgently required. The pathway leading to the conversion of asparagine to isoxazolin-5-one requires further investigation. Elucidation of this pathway may provide an alternative route to eliminating ODAP.
♦ A systematic comparison of CS, CAS, β-(isoxazolin-5-on-2-yl)alanine synthase (BIAS), and DES activities of the BSAS family in *Lathyrus* is required. Is it really the CAS or another one of the BSAS that is dedicated to the BIA function?
♦ The role of protein-protein interaction in cysteine and β-ODAP biosynthesis needs in-depth investigation.
♦ Most modification and regulatory mechanisms in cysteine biosynthesis occur at the post-transcriptional level. Therefore, the role of individual *CS* and *CAS* genes in cysteine and β-ODAP biosynthesis should be assessed carefully, and the regulatory roles of the transcription factors and other critically interacting factors should be investigated.
♦ The development of genomic tools that will accelerate the breeding of β-ODAP-free grass pea lines also needs to be emphasized.

## Abbreviations

BSAS	β-substitute alanine synthase
BIA	β-(isoxazolin-5-on-2-yl)alanine
BIAS	β-(isoxazolin-5-on-2-yl)alanine synthase
CS	Cysteine synthase
CRC	Cysteine regulatory complex
β-ODAP	β-*N*-oxalyl-l-α,β-diaminopropionic acid
β-CAS	β-cyanoalanine synthase
l-/d-DES	l-/d-cysteine desulfhydrase
H_2_S	Hydrogen sulfide
H_2_O_2_	Hydrogen peroxide
ROS	Reactive oxygen species
SiR	Sulfite reductase
SAT	Serine acetyltransferase

## References

[1] Campbell C.G. (1997). Promoting the Conservation and Use of Underutilized and Neglected Crops, 18: Grass Pea, Lathyrus sativus L..

[2] Campbell C.G., Mehra R.B., Agrawal S.K., Chen Y.Z., Abd-El-Moneim A.M., Khawaja H.I.T., Yadav C.R., Tay J.U., Araya W.A. (1994). Current status and future strategy in breeding grass pea (*Lathyrus sativus*). Euphytica.

[3] Kumar S., Bejija G., Ahmed S., Nakkoul H., Sarker A. (2011). Genetic improvement of grasspea for low neurotoxin (β-ODAP) content. Food Chem. Toxicol..

[4] Yan Z.Y., Spencer P.S., Li Z.X., Liang Y.M., Wang Y.F., Wang C.Y., Li F.M. (2006). *Lathyrus sativus* (grass pea) and its neurotoxin ODAP. Phytochemistry.

[5] Rotter R.G., Marquard R.R., Campell C.G. (1991). The nutritional value of low lathyrogenic Lathyrus (*Lathyrus sativus*) for growing chicks. Br. Poult. Sci..

[6] Rao S.L.N., Adiga P.R., Sarma P.S. (1964). The isolation and characterization of β-Noxalyl-l-α,β-diaminopropionic acid, a neurotoxin from the seeds of *Lathyrus sativus*. Biochemistry.

[7] Spencer P.S., Roy D.N., Ludolph A., Hugon J., Dwivedi M.P., Schaumburg H.H. (1986). Lathyrism: Evidence for role of the neuroexcitatory amino acid BOAA. Lancet.

[8] Spencer P.S., Ludolph A.C., Kisby G.E. (1993). Neurologic diseases associated with use of plant components with toxic potential. Environ. Res..

[9] Prakash S., Mishra B.K., Adsule R.N., Barat G.K. (1977). Distribution of β-*N*-oxalyl-l-α,β-diaminopropionic acid in different tissues of aging *Lathyrus sativus* plants. Biochem. Physiol. Pflanz..

[10] Jiao C.J., Xu Q.L., Wang C.Y., Li F.M., Li Z.X., Wang Y.F. (2006). Accumulation pattern of toxin β-ODAP during lifespan and effect of nutrient elements on β-ODAP content in *Lathyrus sativus* seedlings. J. Agric. Sci..

[11] Hussain M., Chowdhury B., Haque R., Lambein F., Haimanot R.T., Lambein F. (1997). Effect of water stress, salinity, interaction of cations, stage of maturity of seeds and storage devices on the ODAP content of Lathyrus sativus. Lathyrus and Lathyrism, a Decade of Progress.

[12] Haque R.M., Kuo Y.H., Lambein F., Hussain M. (2011). Effect of environmental factors on the biosynthesis of the neuro-excitatory amino acid β-ODAP (β-*N*-oxalyl-l-α,β-diaminopropionic acid) in callus tissue of *Lathyrus sativus*. Food Chem. Toxicol..

[13] Roy D.N., Spencer P.S., Cheeke P.R. (1989). Lathyrogens: Toxicants of plant origin. Proteins and Amino Acids.

[14] Haimanot R.T., Kidane Y., Wuhib E., Kalissa A., Alemu T., Zein Z.A., Spencer P.S. (1990). Lathyrism in rural northwestern Ethiopia: A highly prevalent neurotoxic disorder. Int. J. Epidemiol..

[15] Llorens J., Soler-Martín C., Saldaňa-Ruíz S., Cutillas B., Ambrosio S., Boadas-Vaello P. (2011). A new unifying hypothesis for lathyrism, konzo and tropical ataxic neuropathy: Nitriles are the causative agents. Food Chem. Toxicol..

[16] Jiao C.J., Jiang J.L., Ke L.M., Cheng W., Li F.M., Li Z.X., Wang C.Y. (2011). Factors affecting β-ODAP content in *Lathyrus sativus* and their possible physiological mechanisms. Food Chem. Toxicol..

[17] Getahun H., Lambei F., Vanhoorne F., Stuyft M., van der P. (2003). Food-aid cereals to reduce neurolathyrism related to grasspea preparations during famine. Lancet.

[18] Getahun H., Lambein F., Vanhoorne F., Stuyft M., Van der P. (2005). Neurolathyrism risk depends on type of grass pea preparation and on mixing with cereals and antioxidants. Trop. Med. Int. Health.

[19] Kusama-Eguchi K., Yoshino N., Minoura A., Watanabe K., Kusama T., Lambein F., Ikegami F. (2011). Sulfur amino acids deficiency caused by grass pea diet plays an important role in the toxicity of l-β-ODAP by increasing the oxidative stress: Studies on a motor neuron cell line. Food Chem. Toxicol..

[20] Lambein F., Kuo Y.H., Ikegamic F., Kusama-Eguchi K., Enneking D., Kharkwal M.C. (2009). Grain legume and human health. Food Legumes for Nutritional Security and Sustainable Agriculture.

[21] Lambein F., Kuo Y.H., Kusama-Eguchi K., Ikegamic F. (2007). 3-*N*-oxalyl-l-2,3-diaminopropanoic acid, a multifunctional plant metabolite of toxic reputation. ARKIVOC.

[22] La Bella V., Alexianu M.E., Colom L.V., Ionescu A., Mohamed A.H., Appel S. (1996). Apoptosis induced by beta-oxalylamino-l-alanine on a motoneuron hybrid cell line. Neuroscience.

[23] Quereshi M.Y., Pilbeam D.J., Evans C.S., Bell E.A. (1977). The neurolathyrogen, α-amino-β-oxalylaminopropionic acid in legume seeds. Phytochemistry.

[24] Long Y.C., Ye Y.H., Xing Q.Y. (1996). Studies on the neuroexcitotoxin β-Noxalyl-l-α,β-diaminopropionic acid and its isomer α-*N*-oxalo-l-α,β-diaminopropionic acid from the root of *Panax* species. Int. J. Peptide Protein Res..

[25] Pan M.D., Mabry T.J., Cao P., Moini M. (1997). Identification of nonprotein amino acids from cycad seeds as N-ethoxycarbonyl ethyl ester derivatives by positive chemical-ionization gas chromatography-mass spectrometry. J. Chromatogr. A.

[26] Kuo Y.H., Ikegami F., Lambein F. (1998). Metabolic routes of β-(isoxazolin-5-on-2-yl)-l-alanine (BIA), the precursor of the neurotoxin ODAP (β-*N*-oxalyl-l-α,β-diaminopropionic acid), in different legume seedlings. Phytochemistry.

[27] Xie G.X., Qiu Y.P., Qiu M.F., Gao X.F., Liu Y.M., Jia W. (2007). Analysis of dencichine in *Panax notoginseng* by gas chromatography-mass spectrometry with ethyl chloroformate derivatization. J. Pharm. Biomed..

[28] Lambein F., Haque R., Khan J.K., Kebede N., Kuo Y.H. (1994). From soil to brain: Zinc deficiency increases the neurotoxicity of *Lathyrus sativus* and may affect the susceptibility for the motorneurone disease neurolathyrism. Toxicon.

[29] Zhou G.K., Kong Y.Z., Cui K.R., Li Z.X., Wang Y.F. (2001). Hydroxyl radical scavenging activity of β-N-oxalyl-l-α,β-diaminopropionic acid. Phytochemistry.

[30] Zhang J., Xing G.M., Yan Z.Y., Li Z.X. (2003). β-*N*-oxalyl-l-α,β-diaminopropionic acid protects the activity of glycolate oxidase in *Lathyrus sativus* seedlings under high light. Russ. J. Plant Physiol..

[31] Jiang J.L., Su M., Chen Y.R., Gao N., Jiao C.J., Sun Z.X., Li F.M., Wang C.Y. (2013). Correlation of drought resistance in grass pea (*Lathyrus sativus*) with reactive oxygen species scavenging and osmotic adjustment. Biologia.

[32] Jiao C.J., Jiang J.L., Li C., Ke L.M., Cheng W., Li F.M., Li Z.X., Wang C.Y. (2011). β-ODAP accumulation could be related to low levels of superoxide anion and hydrogen peroxide in *Lathyrus sativus* L.. Food Chem. Toxicol..

[33] Campbell C.J., Briggs C.J. (1987). Registration of low neurotoxin content *Lathyrus* germplasm LS8246. Crop Sci..

[34] Abd-El-Moneim A.M., van Dorrestein B., Baum M., Ryan J., Bejiga G. (2001). Role of ICARDA in improving the nutritional quality and yield potential of grasspea (*Lathyrus sativus* L.), for subsistence farmers in dry areas. Lathyrus Lathyrism Newsl..

[35] Pandey R.L., Chitale M.W., Sharma R.N., Geda A.K. (1997). Evaluation and characterization of germplasm of grass pea (*Lathyrus sativus*). JMAPS.

[36] Xing G.S., Cui K.R., Li J., Wang Y.F., Li Z.X. (2001). Water stress and accumulation of β-*N*-Oxalyl-l-α,β-diaminopropionic acid in grass pea (*Lathyrus sativus*). J. Agric. Food Chem..

[37] Xiong J.-L., Xiong Y., Bai X., Kong H., Tan R., Zhu H., Siddique K., Wang J., Turner N.C. (2015). Genotypic variation in the concentration of β-*N*-Oxalyl-l-α,β-diaminopropionic acid (β-ODAP) in Grass Pea (*Lathyrus sativus* L.) seeds is associated with an accumulation of leaf and pod β-ODAP during vegetative and reproductive stages at three levels of water Stress. J. Agric. Food Chem..

[38] Tripathy S.K., Ranjan R., Dash S., Bharti R., Lenka D., Sethy Y.D., Mishra D.R., Mohapatra B.R., Pal S. (2015). Genetic analysis of BOAA content in grasspea (*Lathyrus sativus* L.). Legume Res..

[39] Vaz Patto M.C., Skiba B., Pang E.C.K., Ochatt S.J., Lambein F., Rubiales D. (2006). *Lathyrus* improvement for resistance against biotic and abiotic stresses: From classical breeding to marker assisted selection. Euphytica.

[40] Ikegami F., Itagaki S., Ishikawa T., Ongena G., Kuo Y.H., Lambein F., Murakoshi I. (1991). Biosynthesis of beta-(isoxazolin-5-on-2-yl)alanine, the precursor of the neurotoxic amino acid β-*N*-oxalyl-l-α,β-diaminopropionic acid. Chem. Parm. Bull..

[41] Ikegami F., Itagaki S., Kamiya M., Kuo Y.H., Lambein F., Murakoshi I. (1996). Enzymatic synthesis of two isoxazolylalanine isomers by cysteine synthases in *Lathyrus* species. Biol. Pharm. Bull..

[42] Malathi K., Padmanaban G., Rao S.L.N., Sarma P.S. (1967). Studies on the biosynthesis of β-*N*-oxalyl-l-α,β-diaminopropionic acid, the *Lathyrus sativus* neurotoxin. Biochim. Biophys. Acta.

[43] Malathi K., Padmanaban G., Sarma P.S. (1970). Biosynthesis of β-*N*-oxalyl-l-α,β-diamino-propionic acid, the *Lathyrus sativus* neurotoxin. Phytochemistry.

[44] Kuo Y.H., Lambein F., Mellor L., Adlington R.M., Baldwin J.E. (1994). Ring-nitrogen of β-isoxazolinone-alanine is incorpo- rated into the neurotoxin, β-*N*-oxalyl-l-α,β-diaminopropionic acid, in callus tissue of *Lathyrus sativus*. Phytochemistry.

[45] Kuo Y.H., Lambein F. (1991). Biosynthesis of the neurotoxin β-*N*-oxalyl-l-α,β-diaminopropionic acid in callus tissue of *Lathyrus sativus*. Phytochemistry.

[46] Ikegami F., Horiuchi S., Kobori M., Morishige I., Murakoshi I. (1992). Biosynthesis of neuroactive amino acids by cysteine synthases in *Lathyrus latifolius*. Phytochemistry.

[47] Ikegami F., Ongena G., Sakai R., Itagaki S., Kobori M., Ishikawa T., Kuo Y.H., Lambein F., Murakoshi I. (1993). Biosynthesis of β-(isoxazolin-5-on-2-yl)-alanine, the precursor of the neurotoxin β-*N*-oxalyl-l-α,β-diaminopropionic acid, by cysteine synthase in *Lathyrus sativus*. Phytochemistry.

[48] Machingura M., Salomon E., Jez J.M., Ebbs S.D. (2016). The β-cyanoalanine synthase pathway: Beyond cyanide detoxification. Plant Cell Environ..

[49] Hanbury C.D., White C.L., Mullan B.P., Siddique K.H.M. (2000). A review of the Potential of *Lathyrus sativus* L. and *L. cicera* L. grain for use as animal feed. Anim. Feed. Sci. Technol..

[50] Lambein F. (2009). The Lathyrus/lathyrism controversy. Grain Legumes.

[51] Watanabe M., Kusano M., Oikawa A., Fukushima A., Noji M., Saito K. (2008). Physiological roles of the β-substituted alanine synthase gene family in Arabidopsis. Plant Physiol..

[52] Yi H., Jez J.M. (2012). Assessing functional diversity in the soybean β-substituted alanine synthase enzyme family. Phytochemistry.

[53] Murakoshi I., Kaneko M., Koide C., Ikegami F. (1986). Enzymatic synthesis of the neuroexcitatory amino acid quisqualic acid by cysteine synthase. Phytochemistry.

[54] Ikegami F., Mizuno M., Kihara M., Murakoshi I. (1990). Enzymatic synthesis of the thyrotoxic amino acid mimosine by cysteine synthase. Phytochemistry.

[55] Jiao C.J., Zhao F.Y., Xie S.Q., Yuan J.Y., Yang L.J. (2014). Assay for activities of cysteine synthase and β-cyanoalanine synthase. Amino Acid Biotic Resour..

[56] Wirtz M., Droux M., Hell R. (2004). *O*-acetylserine(thiol)lyase: An enigmatic enzyme of plant cysteine biosynthesis revisited in Arabidopsis thaliana. J. Exp. Bot..

[57] García I., Castellano M.J., Vioque B., Solano R., Gotor C., Romero L.C. (2010). Mitochondrial β-Cyanoalanine synthase is essential for root hair formation in *Arabidopsis thaliana*. Plant Cell.

[58] Romero L.C., Ángeles Aroca M., Laureano-Marín A.M., Moreno I., García I., Gotor C. (2014). Cysteine and cysteine-related signaling pathways in *Arabidopsis thaliana*. Mol. Plant.

[59] Leustek T., Saito K. (1999). Sulfate transport and assimilation in plants. Plant Physiol..

[60] Takahashi H., Saito K. (1996). Subcellular localization of spinach cysteine synthase lsoforms and regulation of their gene expression by nitrogen and sulfur. Plant Physiol..

[61] Heeg C., Kruse C., Jost R., Gutensohn M., Ruppert T., Wirtz M., Hell R. (2008). Analysis of the *Arabidopsis* Oacetylserine(thiol)lyase gene family demonstrates compartment-specific differences in the regulation of cysteine synthesis. Plant Cell.

[62] Hell R., Wirtz M. (2011). Molecular biology, biochemistry and cellular physiology of cysteine metabolism in *Arabidopsis thaliana*. Arabidopsis Book.

[63] Kumaran S., Yi H., Krishnan H.B., Jez J.M. (2009). Assembly of the cysteine synthase complex and the regulatory role of protein-protein interactions. J. Biol. Chem..

[64] Jez J.M., Dey S. (2013). The cysteine regulatory complex from plants and microbes: What was old is new again. Curr. Opin. Struct. Biol..

[65] Yi H., Galant A., Ravilious G.E., Preuss M.L., Jez J.M. (2010). Sensing sulfur conditions: Simple to complex protein regulatory mechanisms in plant thiol metabolism. Mol. Plant.

[66] Yi H., Ravilious G.E., Galant A., Krishnan H.B., Jez J.M. (2010). From sulfur to homoglutathione: Thiol metabolism in soybean. Amino Acids.

[67] Krishnan H.B. (2005). Engineering soybean for enhanced sulfur amino acid content. Crop Sci..

[68] Grossmann K. (1996). A role for cyanide, derived from ethylene biosynthesis, in the development of stress symptoms. Physiol. Plant.

[69] Chivasa S., Carr J.P. (1998). Cyanide restores *N* gene-mediated resistance to tobacco mosaic virus in transgenic tobacco expressing salicylic acid hydroxylase. Plant Cell.

[70] Wong C.E., Carson R.A.J., Carr J.P. (2002). Chemically induced virus resistance in *Arabidopsis thaliana* is independent of pathogenesisrelated protein expression and the *NPR1* gene. Mol. Plant Microbe Interact..

[71] Goudey J.S., Tittle F.L., Spencer M.S. (1989). A role for ethylene in the metabolism of cyanide by higher plants. Plant Physiol..

[72] Gleadow R.M., Møller B.L. (2014). Cyanogenic glycosides: Synthesis, physiology, and phenotypic plasticity. Annu. Rev. Plant Biol..

[73] Yi H., Juergens M., Jez J.M. (2012). Structure of soybean β-cyanoalanine synthase and the molecular basis for cyanide detoxification in plants. Plant Cell.

[74] Bonner E.R., Cahoon R.E., Knapke S.M., Jez J.M. (2005). Molecular basis of plant cysteine biosynthesis: Structural and functional analysis of *O*-acetylserine sulfhydrylase from *Arabidopsis thaliana*. J. Biol. Chem..

[75] Droux M., Ruffet M.L., Douce R., Job D. (1998). Interactions between serine acetyltransferase and *O*-acetylserine (thiol) lyase in higher plants—Structural and kinetic properties of the free and bound enzymes. Eur. J. Biochem..

[76] Berkowitz O., Wirtz M., Wolf A., Kuhlmann J., Hell R. (2002). Use of biomolecular interaction analysis to elucidate the regulatory mechanism of the cysteine synthase complex from *Arabidopsis thaliana*. J. Biol. Chem..

[77] Zhang M.K., Liu F.J., Tao Y.J., Hu X., Li R.S., Xu Q.L. (2016). Cloning of CASase gene from *Lathyrus sativus* and construction of its RNAi vector. Acta Agrestia Sinica.

[78] Das K., Roychoudhury A. (2014). Reactive oxygen species (ROS) and response of antioxidants as ROS-scavengers during environmental stress in plants. Front. Environ. Sci..

[79] Ravindranath V. (2002). Neurolathyrism: Mitochondrial dysfunction in excitotoxicity mediated by L-beta-oxalyl aminoalanine. Neurochem. Int..

[80] López-Martín M.C., Romero L.C., Gotor C. (2008). Cytosolic cysteine in redox signaling. Plant Signal Behavior.

[81] Kopriva S., Mugford S.G., Baraniecka P., Lee B.R., Matthewman C.A., Koprivova A. (2012). Control of sulfur partitioning between primary and secondary metabolism in *Arabidopsis*. Front. Plant Sci..

[82] Álvarez C., Calo L., Romero L.C., Garcıa I., Gotor C. (2010). An Oacetylserine (thiol) lyase homolog with l-cysteine Desulfhydrase activity regulates cysteine homeostasis in *Arabidopsis*. Plant Physiol..

[83] Calderwood A., Kopriva S. (2014). Hydrogen sulfide in plants: From dissipation of excess sulfur to signaling molecule. Nitric Oxide.

[84] Li Z.-G., Min X., Zhou Z.-H. (2016). Hydrogen Sulfide: A Signal Molecule in Plant Cross-Adaptation. Front. Plant Sci..

[85] Chen J., Wu F.H., Wang W.H., Zheng C.J., Lin G.H., Dong X.J., He J.X., Pei Z.M., Zheng H.L. (2011). Hydrogen sulphide enhances photosynthesis through promoting chloroplast biogenesis, photosynthetic enzyme expression, and thiol redox modification in *Spinacia oleracea* seedlings. J. Exp. Bot..

[86] Hancock J.T., Whiteman M. (2014). Hydrogen sulfide and cell signaling: Team player or referee?. Plant Physiol. Biochem..

[87] Hancock J.T., Henson D., Nyirenda M., Desikan R., Harrison J., Lewis M., Hughes J., Neill S.J. (2005). Proteomic identification of glyceraldehyde 3-phosphate dehydrogenase as an inhibitory target of hydrogen peroxide in *Arabidopsis*. Plant Physiol. Biochem..

[88] Lindermayr C., Sallbach G., Durner J. (2005). Proteomic identification of *S*-Nitrosylated proteins in *Arabidopsis*. Plant Physiol..

